# Gastric Adenomas and Mimickers: A Review

**DOI:** 10.3390/diagnostics16121858

**Published:** 2026-06-16

**Authors:** Peter Zanchelli, Krzysztof Glomski, Fleance Gauat, Tony El Jabbour

**Affiliations:** Department of Pathology and Laboratory Medicine, Hartford Hospital, Hartford, CT 06102, USA

**Keywords:** gastric adenomas, gastric polyps, epithelial polyps

## Abstract

Gastric adenomas are epithelial premalignant neoplasms which may appear similar on endoscopic examination; however, histologically, they are classified into four distinct subtypes according to the World Health Organization (WHO) Classification of Tumors of the Digestive System: intestinal adenoma, foveolar-type adenoma, oxyntic gland adenoma, and pyloric gland adenoma. Each subtype has characteristic histologic features that are essential for accurate diagnosis, although distinction can be challenging due to overlapping morphologic patterns. Moreover, these entities differ in their potential for malignant transformation and in their associations with hereditary or syndromic conditions. The objective of this review is to provide a practical guide for the histopathologic diagnosis of gastric adenomas, summarize the evidence regarding their risk of associated malignancy or malignant transformation, and review current recommendations for clinical follow-up and management. Given that various lesions may present endoscopically as gastric polyps, this manuscript also reviews both epithelial and non-epithelial mimickers of gastric adenomas.

## 1. Introduction

Gastric epithelial polyps are lesions that arise from gastric mucosa and include: fundic gland polyps, hyperplastic polyps, and gastric adenomas. Among these entities, gastric adenomas are defined by the presence of epithelial dysplasia, and therefore demonstrate an increased risk of malignant transformation, underscoring their clinical significance [1,2].

Advances in histopathologic classification, along with immunophenotyping and molecular characterization, have refined the distinction between adenomatous and non-adenomatous gastric epithelial polyps, and have improved upon our understanding of their biological behavior and malignant potential [3,4].

This review focuses on gastric adenomas, the prototypical premalignant epithelial lesions of the stomach. To place gastric adenomas in their broader diagnostic context, Section 2 reviews the spectrum of epithelial gastric polyps, including fundic gland polyps, hyperplastic polyps, and gastric adenomas, with emphasis on their classification, histomorphologic features, immunophenotypes, molecular alterations and associated syndromes. Section 3 addresses the clinical management and surveillance of gastric adenomas and related epithelial lesions. Section 4 is dedicated to non-epithelial polypoid lesions that may be present as gastric polyps during endoscopy. Because these lesions comprise a heterogeneous group of entities with distinct genetic origins and histomorphologic features, diagnostic considerations, and management approaches, each entity is discussed separately.

A comprehensive literature search was conducted using electronic databases, such as PubMed, to identify relevant studies published between January 2000 and February 2026, with a select few being published between 1977 and 1999. Search terms included combinations of keywords such as “gastric adenomas,” “intestinal-type gastric adenoma,” “foveolar adenoma,” “pyloric gland adenoma,” “oxyntic gland adenoma,” “gastric polyps,” “epithelial polyps,” “non-epithelial polyps,” “endoscopy,” and “histopathology.” The references cited below were all manually reviewed to identify additional relevant publications. Studies were included if they were original articles, systematic reviews, or clinical guidelines addressing relevant clinical, endoscopic, histological, or management aspects of gastric polyps and their mimickers. Case reports with limited clinical applicability, non-English publications, and studies lacking sufficient methodological details were excluded. Articles were selected based on relevance, scientific quality, and contribution to the scope of this review.

## 2. Pathologic Features of Gastric Epithelial Polyps

### 2.1. Fundic Gland Polyps

**Figure 1:**
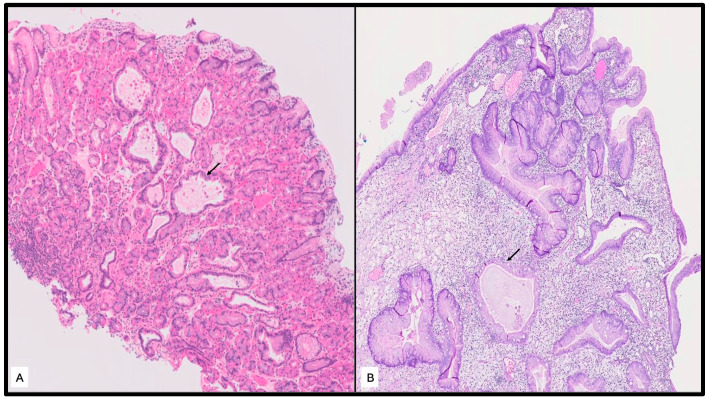
Benign gastric polyps. (**A**) H&E, ×2, showing a fundic gland polyp with cystically dilated oxyntic (fundic) glands (black arrow). (**B**) H&E, ×2, showing a hyperplastic polyp with elongated and cystically dilated foveolar glands (black arrow).

Fundic gland polyps (FGPs) are the most common gastric polyps. They are benign lesions arising from expansion of the deep epithelial (crypt) compartment of the oxyntic (body/fundic) mucosa. Histologically, they demonstrate cystically dilated oxyntic glands lined by parietal cells, chief cells, and occasional mucinous cells (Figure 1A). FGPs most often occur sporadically, often in association with proton pump inhibitor (PPI) therapy, or in the setting of familial adenomatous polyposis (FAP) [5].

Dysplasia within FGPs is rare and, if present, is typically of foveolar-type, composed of low columnar cells resembling foveolar epithelium with round to oval nuclei. Dysplastic FGPs can be difficult to distinguish from foveolar-type adenomas; however, this distinction is of limited clinical importance, as both lesions carry a low risk of progression [5,6].

Although FGPs were traditionally regarded as purely hyperplastic lesions, molecular studies have demonstrated recurrent somatic mutations, supporting a clonal, neoplastic process. Most sporadic non-dysplastic FGPs harbor *CTNNB1* mutations, whereas dysplastic sporadic FGPs more commonly show APC mutations. In FAP-associated FGPs, dysplasia typically follows the somatic second-hit inactivation of APC, occurring in the absence of *CTNNB1* mutations [7,8,9,10].

### 2.2. Hyperplastic Polyps

Hyperplastic polyps are the second most common type of gastric polyp. They are reactive lesions that develop as part of a reparative/regenerative response to mucosal damage, most commonly associated with Helicobacter pylori infection or autoimmune gastritis [11]. Histologically, they are composed of elongated, tortuous, and cystically dilated foveolar glands within an edematous and inflamed lamina propria, which contains numerous capillaries and variably distributed smooth muscle bundles (Figure 1B) [12]. Although hyperplastic polyps are considered benign reactive proliferations, rare instances of associated dysplasia and malignant transformation have been reported [5]. The risk of dysplasia or focal carcinoma within hyperplastic polyps is uncommon, with dysplasia identified in 4% of patients and adenocarcinomas within the polyp in only 0.6% of patients [12].

While hyperplastic polyps are not associated with hereditary syndromes per se, hamartomatous gastric polyps, a rare group of benign neoplastic lesions (with defined genetic alterations), are histologically indistinguishable from hyperplastic polyps and may be encountered in Peutz–Jeghers syndrome, juvenile polyposis syndrome (JPS), and Cowden syndrome (PTEN hamartoma tumor syndrome) [13]. Therefore, an abundance of gastric ‘hyperplastic polyps’ in younger patients may warrant further investigation into the possibility of an underlying syndrome.

### 2.3. Gastric Adenomas

Gastric adenomas are premalignant epithelial neoplasms defined by dysplastic epithelium and an inherent risk of malignant transformation. By convention, adenomas show at least low-grade dysplasia, while the presence of high-grade dysplasia is associated with a higher risk of adenocarcinoma. Gastric adenomas are classified into four major subtypes: intestinal-type adenoma (IGA), foveolar-type adenoma (FGA), pyloric gland adenoma (PGA), and oxyntic gland adenoma (OGA). Each subtype has distinct histologic, immunophenotypic, and clinical characteristics [5,14].

#### 2.3.1. Intestinal-Type Gastric Adenomas

Intestinal-type gastric adenomas (IGAs) are the most common subtype of gastric adenoma and typically arise within intestinal metaplasia, most frequently in the antrum. Predisposing conditions include Helicobacter pylori gastritis and autoimmune gastritis [15], as well as FAP syndrome [10]. Amongst gastric adenomas, intestinal-type adenomas carry the highest risk of malignant progression.

**Figure 2:**
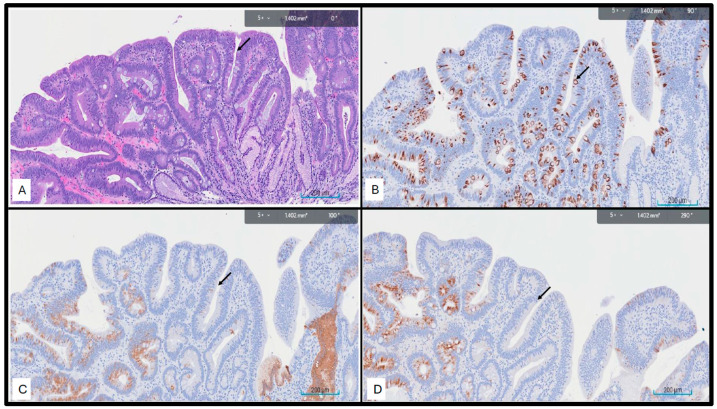
Intestinal-type gastric adenoma. (**A**) H&E, 5×, showing intestinal-type epithelium with intestinal metaplasia and low-grade dysplasia (black arrow). (**B**) MUC2 immunostaining highlighting goblet cells (black arrow). (**C**) MUC5AC immunostaining is negative (black arrow). (**D**) MUC6 immunostaining is negative (black arrow). The combination of MUC2 positivity (within the dysplastic intestinal-type epithelium) with absent MUC5AC and MUC6 is diagnostic of intestinal differentiation and supports the subclassification of IGA.

Histologically, IGAs consist of dysplastic glands demonstrating intestinal differentiation, namely, columnar absorptive epithelial cells, goblet cells, interspersed Paneth and/or endocrine cells. Low-grade dysplasia is characterized by elongated, hyperchromatic, pseudostratified nuclei (Figure 2A), whereas high-grade dysplasia typically shows glandular crowding (with or without cribriform architecture), nuclear stratification (loss of ‘polarity’), and pronounced cytologic atypia. Invasion into the lamina propria or muscularis mucosa defines intramucosal carcinoma, whereas deeper invasion (submucosa, muscularis propria) establishes a diagnosis of invasive adenocarcinoma [16].

Immunophenotypically, IGAs show diffuse MUC2 (and CDX2) expression and apical membranous CD10 staining, consistent with intestinal differentiation, and are typically negative for MUC5AC and MUC6 (Figure 2B–D) [17]. Molecular alterations often include mutations in the *KRAS* and *APC* genes. In a cohort study of 409 intestinal-type gastric adenomas followed longitudinally, 5-year carcinoma development was 34%. Risk factors included lesions greater than 15 mm or central depression. This may not be fully generalized across populations with differing background gastritis [2].

#### 2.3.2. Foveolar-Type Gastric Adenomas

Foveolar-type gastric adenomas (FGAs) are neoplastic polyps composed of dysplastic foveolar epithelium. Unlike intestinal-type adenomas, they arise in the absence of chronic inflammation, atrophy, or intestinal metaplasia, most commonly within the gastric body or fundus [5,6]. These premalignant neoplasms may occur sporadically or in association with FAP and gastric adenocarcinoma and proximal polyposis of the stomach (GAPPS).

**Figure 3:**
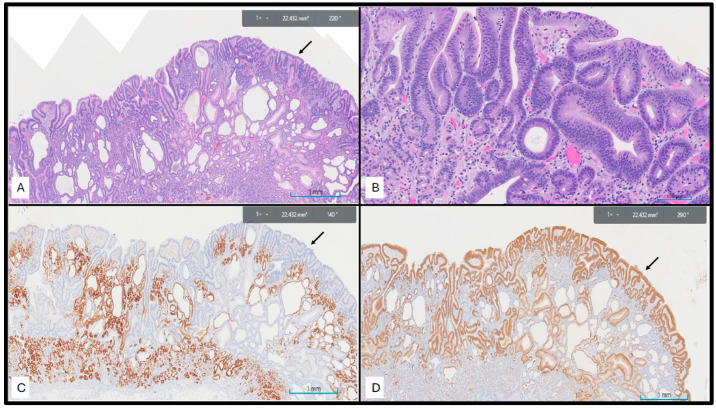
Foveolar-type gastric adenoma. (**A**) H&E, 1×, showing dysplastic foveolar epithelium (black arrow). (**B**) H&E, 5×, high-power view demonstrating penicillate nuclei within the foveolar epithelium and foveolar pits. (**C**) MUC6 immunostaining negative (black arrow). (**D**) MUC5AC immunostaining positive (black arrow). The dysplastic foveolar epithelium with penicillate nuclei that shows diffuse MUC5AC positivity (**D**) and absent MUC6 supports the foveolar phenotype of this gastric adenoma.

Histologically, low-grade FGAs consist of dysplastic columnar epithelium with elongated pseudostratified nuclei, and a characteristic PAS-positive apical mucin cap (Figure 3A,B). High-grade dysplasia is defined by increased glandular crowding, nuclear pleomorphism, hyperchromasia, prominent nucleoli, and cribriform architecture [5,16].

Immunophenotypically, FGAs show diffuse MUC5AC expression, consistent with gastric foveolar differentiation, and lack intestinal markers such as MUC2 and CDX2. MUC6 staining is typically negative, although focal weak positivity may be observed (Figure 3C,D) [17].

FGAs arise in the setting of FAP demonstrate biallelic APC inactivation, whereas sporadic lesions rarely harbor APC or KRAS mutations [4]. The risk of progression to high-grade dysplasia or carcinoma is low in both sporadic and familial foveolar-type adenomas [5,6].

#### 2.3.3. Pyloric Gland Adenomas

Pyloric gland adenomas (PGAs) are neoplastic polyps arising from pyloric-type glands and account for up to 3% of gastric epithelial polyps. Sporadic PGAs are typically observed in patients with conditions that induce pyloric metaplasia, such as autoimmune atrophic gastritis or chronic Helicobacter pylori gastritis. PGAs have also been reported in patients with FAP, where they arise in a non-atrophic background mucosa, as well as in Lynch syndrome, McCune–Albright syndrome, and JPS [18].

**Figure 4:**
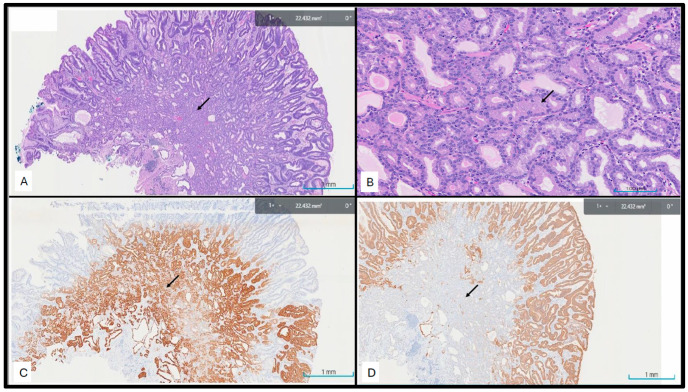
Pyloric gland adenoma. (**A**) H&E, 1×, low-power view showing proliferation of pyloric glands (black arrow). (**B**) H&E, 5×, high-power view demonstrating tightly packed pyloric-type glands lined by cuboidal to low columnar epithelium (black arrow). (**C**) MUC6 immunostaining showing positive results (black arrow). (**D**) MUC5AC immunostaining showing negative results in the adenoma (black arrow), in contrast to positive staining in the overlying foveolar epithelium. The diffuse MUC6 throughout the tightly packed pyloric-type glands (**C**) with MUC5AC restricted to the overlying surface foveolar epithelium (**D**), supports the pyloric phenotype and shows key features distinguishing a PGA from an FGA.

Histologically, PGAs consist of tightly packed pyloric-type glands lined by cuboidal to low columnar epithelium, with glandular dilation often seen in larger lesions. The neoplastic cells show eosinophilic, ground-glass cytoplasm and lack an apical mucin cap (Figure 4A,B). High-grade dysplasia is defined by architectural complexity, nuclear crowding, and loss of cellular polarity [19,20].

Immunohistochemically, PGAs show diffuse MUC6 expression. MUC5AC staining is typically limited to the surface epithelium, although some lesions show diffuse MUC5AC positivity throughout (Figure 4C,D). Intestinal-type markers, including MUC2 and CDX2, are usually negative [19].

PGAs carry a substantial risk of malignant transformation. In a retrospective multicenter study, high-grade dysplasia was found in up to 42% [19], and adenocarcinoma in 12–30% [20]. A hallmark molecular feature is activating GNAS mutations [21].

#### 2.3.4. Oxyntic Gland Adenomas

Oxyntic gland adenomas (OGAs) are rare gastric neoplasms composed predominantly of chief cells and arise in the proximal stomach, typically in the absence of oxyntic mucosal atrophy or Helicobacter pylori infection [22,23,24].

**Figure 5:**
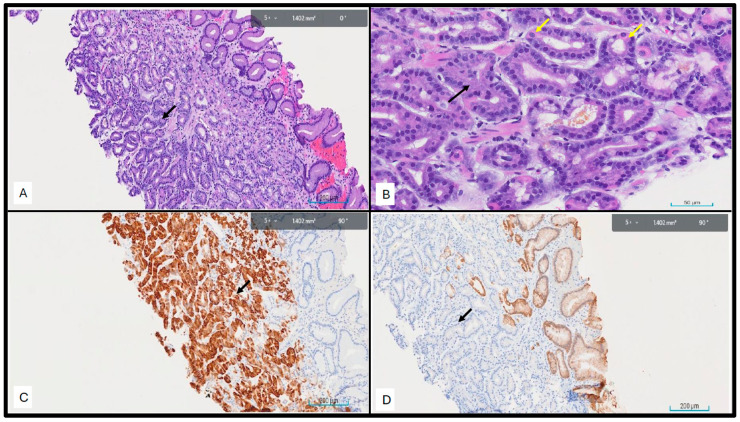
Oxyntic gland adenoma. (**A**) H&E, 5×, low-power view showing proliferation of oxyntic glands (black arrow). (**B**) H&E, 10×, high-power view showing irregular glands composed predominantly of chief cells with basophilic cytoplasm and mild nuclear atypia (black arrow), with intermixed parietal cells (yellow arrow). (**C**) MUC6 immunostaining positive in the adenoma (black arrow). (**D**) MUC5AC immunostaining negative in the adenoma (black arrow), in contrast to positive staining in the overlying foveolar epithelium. The irregular glands composed of predominantly chief cells show diffuse MUC6 staining (**C**) with retained MUC5AC (**D**) only in the overlying foveolar epithelium is supportive of the phenotype of OGA.

Histologically, OGAs consist of irregularly arranged oxyntic glands primarily composed of chief cells with basophilic cytoplasm and mild nuclear atypia. Lesions often show tubular fusion and lateral expansion deep within the mucosa and are covered by non-neoplastic foveolar epithelium. Some cases contain intermixed parietal cells (Figure 5A,B) [22,23,24].

Immunophenotypically, OGAs reflect chief cell differentiation, showing diffuse pepsinogen I positivity and MUC6 expression, while MUC5AC, CDX2, and MUC2 are typically negative (Figure 5C,D) [22,23,24,25,26,27].

Most lesions are restricted to the mucosa and demonstrate only mild atypia, consistent with benign behavior. When submucosal invasion is present, the lesion is classified as gastric adenocarcinoma of fundic gland type, which generally shows a pushing growth pattern without a desmoplastic stromal reaction. Despite occasional submucosal involvement, the malignant potential of OGAs is very low, with lymph node metastasis being extremely rare, even in cases with submucosal or lymphovascular invasion.

Molecular studies have identified *GNAS* mutations in OGAs, supporting a potential relationship between PGAs and OGAs as part of a shared neoplastic spectrum [28,29,30,31].

## 3. Clinical Management and Surveillance

Hereditary syndromes associated with gastric epithelial polyps include FAP, characterized by FGPs, FGAs, and PGAs; GAPPS, characterized by numerous proximal FGPs with foveolar dysplasia; and Lynch syndrome, which is associated with an increased risk of gastric cancer despite often lacking significant gastric polyposis. Histology, particularly the presence of dysplasia, remains the primary determinant of malignant risk across all syndromes [18].

The clinical management for gastric epithelial polyps, according to the NCCN guidelines of Genetic/Familial High-Risk Assessment: Colorectal, Endometrial, and Gastric Guidelines (updated June 2025), includes complete endoscopic resection. Following endoscopic resection, surveillance intervals are recommended depending on several factors, such as histology with dysplastic features, polyp size and completeness of resection. The primary aim of these recommendations is the detection of synchronous and metachronous lesions and the prevention of progression to gastric adenocarcinoma. In addition, the guidelines provide standardized, evidence-based surveillance intervals for patients with FAP, reducing reliance on expert opinion [18,32,33,34].

The management of FGPs and hyperplastic polyps differs from gastric adenomas. Fundic gland polyps typically do not require routine surveillance, especially in cases without dysplasia and not associated with FAP, as these polyps harbor low malignant potential. If these are associated with proton pump inhibitors (PPI), then discontinuation of PPIs can lead to regression [35,36,37]. However, the clinical decision to discontinue PPIs solely for the purpose of promoting FGP regression remains controversial, given the benign nature of most FGPs and the frequent need for ongoing acid suppression. Consequently, PPI cessation is generally not recommended unless the underlying indication for therapy is uncertain. In FGPs associated with FAP, there is an increased risk for malignant potential [36]. In these patients, surveillance is stratified by size (<1 cm: 3 years; ≥1 cm: 1 year), with intervals shortened to 3–6 months in the presence of high-grade dysplasia [18]. Hyperplastic polyps are typically resected when >2 cm in size due to increased malignant potential. After resection, underlying etiologies such as Helicobacter pylori infection and autoimmune gastritis should be addressed [11,12,15,37].

For neoplastic polyps, such as the IGAs and PGAs, there is high risk for malignant potential; therefore, complete endoscopic resection is crucial. IGAs with high-grade dysplasia warrant endoscopic submucosal dissection (ESD) to assess for intramucosal carcinoma [33,38]. Metachronous lesions can be prevented by evaluating for Helicobacter pylori within the first 6 to 12 months post-resection, then after longer intervals in the absence of recurrence [32]. PGAs are strongly associated with high-grade dysplasia or carcinoma and are typically followed annually, with this timeframe being shortened to 3–6 months after high-grade dysplasia or piecemeal resection. Monitoring for autoimmune gastritis is also recommended [19,20,21].

FGAs and OGAs demonstrate limited biologic potential, warranting less aggressive surveillance, although complete endoscopic resection is recommended. FGAs (sporadic or FAP-associated) follow the same principles as FGPs. OGAs may rarely show high-grade dysplasia or submucosal (pushing) invasion but have low recurrence and progression risk; thus, resection is generally curative [39,40].

Among endoscopic removal techniques, the two most prominent are endoscopic mucosal resection (EMR) and endoscopic submucosal dissection (ESD). While both may address lesions with high-grade dysplasia, EMR is typically reserved for smaller lesions without intramucosal or submucosal invasion. EMR uses snares to resect at the mucosal surface, which is limited to en bloc resections of less than 20 mm [33,38,40]. In contrast, ESD utilizes electrosurgical knives to dissect into the submucosa to completely resect en bloc without size restriction.

In terms of oncologic outcome, ESD is the superior technique throughout the gastrointestinal tract. With gastric lesions specifically, the clinical success rates were 86.5% for ESD and 54.5% for EMR, while local recurrence rates were 1.7% and 7.2%, respectively. Although ESD has better clinical success and lower local recurrence, it has a higher rate of adverse events, including bleeding, perforation, and scarring/stricture. The perforation rates for EMR and ESD are 1.9% and 3.7%, respectively [38,39,40]. These outcomes are pooled from meta-analyses comparing EMR and ESD for gastric neoplastic lesions, and heterogeneity in lesion size, location, and operator expertise across the included studies should be considered. Practically, EMR remains an appropriate choice for small (<15–20 mm), well-circumscribed lesions confined to the mucosa with low-grade dysplasia. In contrast, ESD is preferred for larger lesions (>20 mm), lesions with high-grade dysplasia or suspected intramucosal carcinoma, and any lesion for which en bloc resection with surgical margin evaluation is required for accurate pathologic staging.

The histologic characteristics, ancillary studies, management and syndromic associations of gastric adenomas are summarized in Table 1.

## 4. Gastric Non-Epithelial Lesions—Polypoid Mimics

Several non-epithelial lesions may present as polypoid gastric lesions and enter the differential diagnosis of gastric epithelial polyps. This section focuses on their endoscopic appearance, key histopathologic and immunohistochemical features, and management considerations relevant to their polypoid presentation.

### 4.1. Gastrointestinal Stromal Tumors (GIST)

Gastrointestinal stromal tumor (GIST) can occur anywhere within the gastrointestinal (GI) tract. These are most often found in the stomach (up to 70% of all GISTs) and are the most common mesenchymal neoplasm of the stomach, originating from the interstitial cells of Cajal. Their behavior varies between benign and aggressive, and their size varies from incidental nodules to large tumors [41]. GISTs are non-epithelial (intramural) lesions compared to true mucosal polyps, with up to 25% being incidental findings on imaging or upper endoscopy. Clinical presentation varies from asymptomatic to abdominal pain, GI bleeding, and ulcers [42]. Endoscopic findings of GISTs are characterized as smooth, round, submucosal bulges with normal overlying mucosa, sometimes with central ulceration. Endoscopic ultrasound (EUS) is typically used to characterize which type of non-epithelial lesion is being seen. Typically, a hypoechoic mass arises from the fourth layer (muscularis propria) on the EUS [43]. Features suggestive of high risk on EUS include irregular borders, ulceration, cystic spaces, and heterogeneity. The most important features predicting malignant potential are irregular borders and size > 2 cm [1]. Histologically, these can present with a wide spectrum of morphology, with variation between gastric and small bowel GISTs. Most commonly, gastric GISTs are composed of spindle cell neoplasms, with an epithelioid appearance in up to 25% of cases. Nuclear pleomorphism is not common in these neoplasms. There is also a mixed variant with a combined morphology of both spindle and epithelioid components. Among the spindle cell predominant GISTs, there are distinct histological patterns that exist. The sclerosing type is a subtype that contains calcifications in smaller tumors. One of the more common subtypes is the palisaded-vacuolated subtype, which presents with a diffuse hypercellular pattern. More rarely, sarcomatoid features (e.g., significant nuclear atypia with high mitotic activity) may be observed. In contrast, epithelioid GISTs sometimes show a pseudopapillary pattern with hypercellularity. Also seen are the sclerosing, discohesive variants and, rarely, GISTs with myxoid stroma [44]. GISTs commonly show an immunohistochemical staining with strong and diffuse CD117 (KIT) and DOG1 expression, although, very rarely, those with PDGFRA mutations may lack CD117 expression [45]. The normal interstitial cells of Cajal also stain positive for DOG1/CD117, further supporting the origin of GISTs. Gastric GISTs (spindle cells) may also stain positive for CD34, whereas the epithelioid-predominant GISTs are not reliably positive [46]. Most GISTs are sporadic, but fewer than 10% have syndromic associations. Most of the associations are succinate dehydrogenase (SDH)-deficient, including the non-hereditary Carney Triad (GIST, pulmonary chondroma, paraganglioma) and autosomal dominant Carney–Stratakis syndrome (GIST and paraganglioma) [47,48]. The most common mutation seen (85% of lesions) is gain-of-function in KIT or PDGFRA oncogene on chromosome 4, which encodes the type III tyrosine kinase receptor. Approximately 75% of GISTs have activating KIT mutations within exon 11 and 9. Less than 1% have KIT mutations in exon 13 and 17 [49]. The initial management of gastric GISTs is primarily determined by their size as assessed endoscopically. If there are no high-risk features and <2 cm, then an EUS-guided tissue sample is warranted, along with periodic surveillance. If high-risk features on EUS or on biopsy are seen with minimal morbidity, then complete surgical resection via wedge resection is sufficient. When the lesion shows significant morbidity but is still resectable, targeted tyrosine kinase inhibitors are recommended. In the more severe scenarios, where the lesion is unresectable or with metastatic disease, lifelong tyrosine kinase inhibitors are warranted. Prognosis and malignant potential are determined by the size of the tumor, mitotic activity per 50 high-power fields (HPFs), and anatomic location. Gastric GISTs typically have the more favorable prognosis when compared to other anatomic sites. SDH-deficient GISTs are typically more unpredictable and standard guidelines cannot be relied on to predict prognosis [50].

### 4.2. Leiomyoma

Gastric leiomyomas are rare, benign mesenchymal neoplasms of the smooth muscle cells. These typically are asymptomatic with an insidious onset but can present with symptoms such as bleeding/ulceration, anemia, abdominal pain or dyspepsia. On imaging, they tend to mimic other gastric neoplasms, including GISTs, which can lead to misdiagnosis or sometimes unnecessary interventions. Leiomyomas arise from the smooth muscle cells within the muscularis mucosae or propria and make up a small portion of non-epithelial lesions of the stomach. These are usually incidentally found during endoscopy or surgery, presenting as a polyp [51]. The most common location within the stomach is the cardia (77%), and they average 3 cm in size [52]. During endoscopy, intact normal mucosa with an underlying smooth submucosal bulge is seen, which is a nonspecific finding. During EUS, there is a homogenous, hypoechoic lesion arising from either the second, third, or fourth layer; considering this is nonspecific, tissue diagnosis is necessary for confirmation [41]. Microscopically, these tumors are composed of bundles of spindled smooth muscle cells with blunt-ended nuclei and eosinophilic cytoplasm that may be arranged in fascicles or whorls. Mitotic activity is typically low in this benign entity [53]. These can be difficult to distinguish from a GIST if they are colonized by interstitial cells of Cajal. A pitfall is that these colonized interstitial cells of Cajal may stain positive with CD117/DOG1, mimicking a GIST, especially on biopsy. The typical immunoprofile seen in this entity are positive stains for desmin, smooth muscle actin, caldesmon, and calponin. Gastric leiomyomas do not have clinically relevant molecular alterations and lack the KIT/PDGFR mutations seen in GISTs [41,54]. Gastric leiomyomas specifically are not associated with specific syndromes, although gastrointestinal leiomyomas may be germline and associated with Alport syndrome with diffuse leiomyomatosis, with genetic analysis showing deletion of COL4A5/COL4A6 genes [55]. Clinical management for asymptomatic patients and histologically proven leiomyoma involves close monitoring with regular follow-up, which is sufficient due to their excellent prognosis. Resection of this lesion is recommended if patients are symptomatic, if the tumor is ulcerated or if there is a size increase [52].

### 4.3. Schwannoma

Gastric schwannomas are rare, benign non-epithelial tumors originating from the Schwann cells in the gastrointestinal autonomic nervous plexus. The most common site within the GI tract is within the stomach (mostly gastric body), representing up to 90% of GI-related schwannomas, and they are often misdiagnosed as GISTs. Schwannomas have a female predominance in patients older than 50 years. They may present asymptomatically and are often found incidentally during endoscopy. When symptomatic, patients experience bleeding, abdominal pain, or discomfort, especially if there is a sizeable tumor causing outlet obstruction [56]. During endoscopy, these appear as submucosal bulges with unremarkable overlying mucosa. On EUS, this demonstrates a nonspecific hypoechoic lesion arising from the muscularis propria (fourth layer) that is a well-demarcated, homogenous mass and rarely shows cystic changes or calcifications [57]. When tissue diagnosis is obtained, typical histological findings include an unencapsulated, well-circumscribed nodule confined to the muscularis propria. These tumors have characteristic spindle cells in a microtrabecular architecture with focal nuclear atypia and peritumoral lymphoid cuffs containing germinal centers. The microcystic/reticular subtype is a differentiation seen in GI schwannomas compared to soft tissue or CNS schwannomas (which lack Antoni A/B patterns with nuclear palisading). This subtype has a microcystic and reticular growth pattern with intersecting strands of spindle cells that display eosinophilic cytoplasm within hyalinized or myxoid stroma, with a low mitotic count. The immunohistochemical profile for this entity is strongly and diffusely positive for S100, and most are also positive for GFAP. In contrast to other non-epithelial lesions, these are negative for HMB45, CD117, DOG1, SMA, desmin and synaptophysin [58,59]. Conventional schwannomas are associated with neurofibromatosis 2, with loss of heterozygosity at NF2 mutations which lead to loss of merlin expression. NF2-driven tumorigenesis (biallelic NF2 inactivation) is the pathway that conventional schwannomas follow. In contrast, GI schwannomas do not follow the NF2-driven tumorigenesis pathway. This distinct molecular profile correlates with the distinct histological findings of GI schwannomas, suggesting an alternative pathogenesis [60]. These tumors follow a benign clinical course without any reported malignant subtypes and are resected if symptomatic, typically by wedge gastrectomy in larger tumors. Schwannomas have an excellent prognosis with no local or residual recurrence of the tumor [57].

### 4.4. Lipoma

Lipoma is a benign non-epithelial tumor composed of mature adipose tissue, which represent < 3% of all benign gastric neoplasms. These most commonly present in the antrum, followed by the body of the stomach. The majority are asymptomatic and are found incidentally in patients within their sixth decade of life. These tumors can cause symptoms if large (>4 cm) and present with GI bleeding, abdominal pain and gastric outlet obstruction [61]. Unlike other non-epithelial lesions, lipomas have distinct endoscopic and radiological findings. Radiological findings reveal a well-circumscribed, homogenous mass with fat attenuation; this is diagnostic in 95% of cases. Endoscopic findings reveal a yellow hue visible through the overlying mucosa, despite being a submucosal lesion. During the procedure, endoscopists can perform the “pillow sign,” which is a soft indentation of the tumor when pressed with cold forceps; this is 98% specific with 40% sensitivity. In ulcerated lesions, “tenting” of the fat may be seen. Routine mucosal biopsies are not deep enough to render a diagnosis of these submucosal tumors. In 80% of cases during EUS, there is a well-defined lesion that is homogenous and hyperechoic, arising from the third layer (submucosa) [62,63]. When tissue sampling is obtained, microscopic examination reveals mature adipocytes with uniform size and minimal cytological atypia. Immunohistochemistry stains are typically not required, as this is a hematoxylin and eosin diagnosis [43]. Tissue sampling is rarely needed because lipomas have distinct endoscopic and radiologic features [63]. Resection is only warranted if lipomas become symptomatic. Endoscopic resection (ESD, EMR) is sufficient if the lipoma is ≥2 cm, with wedge resections reserved for very large lesions [64]. Lipomas, because of their benign nature, have an excellent prognosis and do not require surveillance [41].

### 4.5. Inflammatory Fibroid Polyp

Inflammatory fibroid polyps (IFPs) are reactive, non-neoplastic, non-epithelial polyps that arise from the submucosa but may protrude into or involve the overlying mucosa [65]. These polyps are most common in the gastric antrum, with a female predominance in middle-aged adults. There are no clearly associated risk factors for IFPs, but some cases have reported Helicobacter pylori infection as a potential risk factor. Helicobacter pylori has been found to be present in approximately 48% of gastric IFPs. A significant finding in all the cases is chronic gastritis, including intestinal and foveolar hyperplasia [66]. Patients are typically asymptomatic, although when symptoms occur, they present with abdominal pain and GI bleeding [67]. These polyps are commonly found incidentally as semi-pedunculated protrusions with unremarkable overlying mucosa. Polyps larger than 1 cm may have central erythematous depression, ulceration, and bridging folds. They are typically non-diagnostic on biopsy, and endoscopic resection is needed for definitive diagnosis. During EUS, these present with hypoechoic to hyperechoic lesions with indistinct margins and a homogenous appearance that originate from the submucosa or muscularis propria (third or fourth layer, respectively). When a tissue sample is obtained, histologically they present as submucosal lesions with mucosal extension and spindle cells arranged perivascularly in an “onion skin” pattern. However, if these polyps are associated with Helicobacter pylori infection, these cases less frequently demonstrate the onion-skin features. A key feature is eosinophilic-rich infiltrate [43,66,68]. The immunohistochemical profile shows positivity for CD34 and PDGFRA in most cases. These polyps are negative for CD117/DOG1, S100, and desmin [67,68,69]. Most of these polyps are sporadic but can rarely be familial, with associated PDGFRA germline mutations. This entity, when associated with germline mutations, was formerly termed intestinal neurofibromatosis and was included within the familial GISTs due to similar genotypes with PDGFRA-mutant gastrointestinal tumor [70]. Clinical management of these benign lesions involves snare polypectomy by endoscopic resection, which has an excellent prognosis and no risk of recurrence [66].

## 5. Conclusions and Future Directions

Gastric adenomas represent a heterogeneous group of premalignant lesions with distinct histopathologic, immunophenotypic, and molecular features that directly influence malignant potential and clinical management. Accurate classification is essential for stratifying risk, guiding endoscopic resection strategies, and determining surveillance intervals. Ongoing advances in molecular characterization continue to refine the understanding of these epithelial precursor lesions and support tailored management approaches aimed at preventing progression to gastric adenocarcinoma. Distinguishing adenomatous lesions from non-epithelial lesions that present with a polypoid appearance during endoscopy is an important diagnostic consideration when evaluating gastric polyps.

Several outstanding questions remain to be addressed. The role of next-generation sequencing in the routine subclassification of gastric adenomas, particularly the morphologic overlap between foveolar, pyloric, and oxyntic gland lesions, has not yet been fully established. Likewise, the integration of artificial intelligence into digital pathology and higher-definition endoscopy may enhance lesion detection and potential risk stratification, although prospective validation studies are still needed. Additionally, investigation of the signaling pathway regulators involved in the adenoma-to-carcinoma transition may yield clinically useful biomarkers. Raf-kinase inhibitor protein (RKIP), an endogenous suppressor of the Raf/MEK/ERK cascade, exhibits progressive loss across the gastric carcinogenesis spectrum and is directly degraded by Helicobacter pylori through CagA-dependent mechanisms [71]. Given that KRAS mutations (the same pathway that RKIP inhibits) have been identified as late-stage events in gastric adenomas [4], RKIP expression warrants direct investigation in gastric adenoma tissue as a potential marker for transformation risk. Finally, as molecular markers associated with low biological aggressiveness become better validated, surveillance strategies may safely be deescalated for selected low-risk lesions, such as small oxyntic gland adenomas and sporadic foveolar-type adenomas.

Several controversies remain unresolved, including interobserver variability in dysplasia grading, the absence of standardized surveillance intervals following piecemeal resection, and the uncertain management of incidentally detected subcentimeter (<1 cm) oxyntic gland lesions, where the threshold between surveillance and intervention is not well established.

## Figures and Tables

**Table 1:** Comprehensive comparison of the four subtypes of gastric adenomas. * Features of high-grade dysplasia: crowded glands, loss of polarity, and cribriform architecture. FAP: Familial Adenomatous Polyposis; GAPPS: gastric adenocarcinoma and proximal polyposis of the stomach; IGA: intestinal-type gastric adenoma.

Features	IGA (Intestinal-Type)	FGA (Foveolar-Type)	PGA (Pyloric Gland)	OGA (Oxyntic Gland)
Typical location	Antrum	Body/fundus (within FAP/GAPPS)	Body, fundus, antrum	Body/fundus
Background Mucosa	Intestinal metaplasia due to autoimmune gastritis or *H. pylori* infection	Normal mucosa	Autoimmune gastritis and intestinal metaplasia; normal background mucosa when associated with FAP, Lynch Syndrome	Normal oxyntic mucosa
Key Histological Features	Intestinal-type epithelium with pseudostratified nuclei, goblet cells, Paneth cells and ±high-grade dysplasia *	Columnar foveolar epithelium with apical mucin cap (elongated pseudostratified nuclei) ±high-grade dysplasia *	Tightly packed pyloric-type glands lined by columnar to cuboidal cells, lacking apical mucin cap, eosinophilic ground glass cytoplasm ± high-grade dysplasia *	Oxyntic glands with basophilic chief cells, some parietal cells with mild atypia
Immunohistochemistry	MUC2+, CD10+, CDX2+, MUC5AC−, MUC6−	MUC5AC+, variable MUC6; MUC2−, CDX2−, CD10−	MUC6+ (diffuse), MUC5AC+ (coexpression); CDX2−, MUC2−	Pepsinogen-I + (chief cells), MUC6+; MUC5AC−, MUC2−, CDX2−
Typical Molecular Alterations	APC, KRAS	APC, KRAS	GNAS	GNAS
Malignant Risk	High: 34% 5-year carcinoma development	Low: high-grade dysplasia and carcinoma rare in sporadic cases; higher risk in FAP/GAPPS	High: high-grade dysplasia up to 42%; adenocarcinoma risk between 12 and 30%	Low: rarely submucosal invasion
Recommended Surveillance	Complete ESD to assess for high-grade dysplasia; Endoscopy at 6–12 months	Same as IGA	Same as IGA/FGA	Same as IGA/FGA/PGA; endoscopic resection usually curative
Syndromic Associations	FAP	FAP and GAPPS	FAP and Lynch Syndrome	Arises sporadically without known syndromic association

## Data Availability

The original contributions presented in this study are included in the article. Further inquiries can be directed to the corresponding author.

## Abbreviations

AGA, American Gastroenterological Association; ACG, American College of Gastroenterology; AIG, autoimmune gastritis; ASGE, American Society for Gastrointestinal Endoscopy; CDX2, caudal-type homeobox 2; EMR, endoscopic mucosal resection; ESD, endoscopic submucosal dissection; EUS, endoscopic ultrasound; FAP, familial adenomatous polyposis; FGA, foveolar-type gastric adenoma; FGP, fundic gland polyp; GAPPS, gastric adenocarcinoma and proximal polyposis of the stomach; GIST, gastrointestinal stromal tumor; HGD, high-grade dysplasia; IFP, inflammatory fibroid polyp; IGA, intestinal-type gastric adenoma; JPS, juvenile polyposis syndrome; LGD, low-grade dysplasia; MUC, mucin; NCCN, National Comprehensive Cancer Network; OGA, oxyntic gland adenoma; PGA, pyloric gland adenoma; PPI, proton pump inhibitor; SDH, succinate dehydrogenase; WHO, World Health Organization.
